# Effectiveness of Narrowband Ultraviolet Light in Chronic Kidney Disease-Associated Pruritus

**DOI:** 10.7759/cureus.53340

**Published:** 2024-01-31

**Authors:** Imane Shabi, Zineb Aboudar, Mounia Sidki, Said Amal, Ouafa Hocar, Maryem Aboudourib, Nabil Hamouche, Mariam Chettati, Wafaa Fadili, Inass Laouad

**Affiliations:** 1 Nephrology, Mohammed VI University Hospital, Marrakesh, MAR; 2 Dermatology, Mohammed VI University Hospital, Marrakesh, MAR

**Keywords:** chronic kidney disease (ckd), nb-uvb, dialysis, phototherapy, uremic pruritus

## Abstract

Introduction: Chronic kidney disease-associated pruritus (CKDaP) is a prevalent and challenging symptom in individuals suffering from advanced chronic kidney disease (CKD). Its underlying mechanism remains inadequately understood, leading to a limited array of unsatisfactory therapeutic interventions. Despite various attempts, identifying the most effective treatment remains inconclusive. Nevertheless, there is a growing interest in employing ultraviolet phototherapy, particularly for non-responsive patients, although its efficacy is not definitively established. To investigate the potential benefits of narrowband ultraviolet B (NB-UVB) phototherapy on individuals experiencing CKDaP, we report our experience with NB-UVB light in management of CKDaP in dialysis patients.

Materials and methods: The study group consisted of patients with end-stage chronic kidney disease who underwent hemodialysis. These patients received dermatological consultations and follow-ups for itching. They were all unresponsive to the conventional treatment (emollients and antihistamines). Screening laboratory examinations, including complete blood count, liver function test, thyroid function, electrolytes, and others, were also arranged to exclude systemic etiologies. The main potential pruritogens were dosed: calcium, phosphate, and parathyroid hormone. Itch intensity was evaluated with a numerical rating scale (0-10), based on the worst level of itching in the past two weeks. They had sessions of NB-UVB light (311 nm, TL01) twice per week. After UVB exposure, patients were advised to use topical emollients. A questionnaire was employed to document the extent, intensity, frequency, and sleep disruption experienced to evaluate the efficiency of the treatment, using a scale from 0 to 10.

Results: In a group of 38 patients, the average age of the patients was 56 years (16-80); 63.2% were female and 36.8% were male. Median duration of pruritus was 4.7 years, and that of dialysis was 8.4 years. Pruritus was intermittent and diffuse in most cases, localized to the arteriovenous fistula site in two cases, and exacerbated by heat in all cases. Itch intensity was evaluated with a numerical rating scale (0-10) based on the worst level of itching in the past two weeks and showed a moderate average score (5/10). Xerosis was found in 63%, and scratch lesions such as excoriation in 34%. NB-UVB phototherapy was used twice per week on nonconsecutive days, with protection of the genital area and also the eyes using UVB-blocking goggles. The initial dose was 0.4 J/cm^2^ and further doses were introduced according to the erythema response until a maximum of 2 J/cm^2^. No sunburn, hyperpigmentation, or blistering was noted. Emollients were maintained in patients with xerosis. Average number of sessions was 13 (6-24) and reduction of itch intensity was observed starting from the sixth session. Total improvement was obtained at the end of treatment duration except for three patients who required additional sessions. One patient had recurrence one year later.

Conclusion: In conclusion, phototherapy represents a significant advancement in the treatment options for CKD-associated pruritus. Its positive impact on reducing itching and improving the quality of life for many patients is undeniable. However, to fully unlock its potential, ongoing research is needed to optimize dosing, understand relapse mechanisms, and identify the patients who will benefit most from this therapy.

## Introduction

Chronic kidney disease-associated pruritus (CKDaP) is a prevalent and challenging symptom in individuals suffering from advanced chronic kidney disease. Its underlying mechanism remains inadequately understood, leading to a limited array of unsatisfactory therapeutic interventions. Managing uremic pruritus predominantly involves utilizing emollients and systemic treatments like oral antihistamines, gabapentin, doxepin, naloxone, and naltrexone as a primary approach. Despite various attempts, identifying the most effective treatment remains inconclusive. Nevertheless, there is a growing interest in employing ultraviolet phototherapy, particularly for non-responsive patients, although its efficacy is not definitively established [1,2].

Gilchrest et al. was the first to investigate the use of ultraviolet B light (UVB) for uremic pruritus in a controlled clinical trial. Their findings showed some improvement in pruritus among a subset of patients [3-6]. Subsequently, Tan et al. conducted a meta-analysis in 1991, concluding that UVB phototherapy exhibited significant clinical efficacy for treating uremic pruritus. Diverse forms of ultraviolet light, such as UVA, broad band ultraviolet B (BB-UVB), and narrowband ultraviolet B (NB-UVB), have been employed. The precise mechanism underlying the alleviation of pruritus by NB-UVB is not entirely elucidated but is believed to involve various effects on cell cycle kinetics, changes in cytokine expression, impacts on mast cells, and immunomodulation. Additionally, NB-UVB is suggested to directly influence divalent ions, suppress histamine release from cutaneous mast cells, and modify cutaneous nerves [4-11].

Research on narrowband ultraviolet B (NB-UVB) phototherapy for chronic kidney disease-associated pruritus (CKDaP) is constrained [4]. Furthermore, the efficacy and safety of NB-UVB treatment for patients with chronic kidney disease-associated pruritus (CKDaP) and Fitzpatrick skin phototypes 3-5 have not been extensively studied. This study aims to evaluate the application of NB-UVB phototherapy in alleviating chronic kidney disease-associated pruritus (CKDaP) among patients with Fitzpatrick skin phototypes 3-5.

To investigate the potential benefits of narrowband ultraviolet B phototherapy on individuals experiencing chronic kidney disease-associated pruritus (CKDaP), we report our experience with narrowband ultraviolet B (NB-UVB) light in management of chronic kidney disease-associated pruritus (CKDaP) in dialysis patients.

This article was previously presented as a meeting abstract at the 2023 EADV Annual Scientific Meeting on April 11 October, 2023.

## Materials and methods

The study group consisted of 38 patients with end-stage chronic kidney disease who underwent hemodialysis at the Ibnoutoufail Center in Marrakech between 2017 and 2022. These patients received dermatological consultations and follow-ups for itching. They were all unresponsive to the conventional treatment (emollients and antihistamines).

Patients meeting any of the following criteria were excluded from the study: those with alternative dermatological causes of pruritus, individuals with underlying systemic conditions that could induce pruritus, patients with parathyroid hormone levels exceeding 520 pg/mL or serum calcium levels over 10.2 mg/dL, pregnant or breastfeeding women, and those with a history of cutaneous photosensitivity, eye cataracts, or skin cancer.

Each patient underwent a comprehensive evaluation, including a detailed medical history, to gather information on the onset and duration of pruritus, its characteristics (constant or intermittent), severity, factors that worsened or alleviated it, and any prior treatments used. A thorough physical and dermatological examination was conducted, including an assessment of the patient's Fitzpatrick skin type.

Screening laboratory examinations, including complete blood count, liver function test, thyroid function, and electrolytes, were also arranged to exclude other systemic etiologies. The main potential pruritogens were dosed: calcium, phosphate, creatine phosphokinase (CPK), lactic acid dehydrogenase (LDH), and parathyroid hormone. The dialysis efficacy was re-evaluated to increase efficiency and correct electrolytes in cases of imbalance. Diet adjustment with low protein and probiotics also served as potential treatment strategies.

Once an accurate diagnosis of uremic pruritus is made, physicians prescribe an emollient for skin rehydration. Itch intensity was evaluated with a numerical rating scale (0-10) based on the worst level of itching in the past two weeks. Mild itching was rated 0 to 3, moderated 4 to 6, and severe 7-10.

The patients were provided with a comprehensive explanation of the treatment's nature, duration, potential side effects, and possible complications. Informed consent was obtained from all participants before the start of the treatment. They had sessions of NB-UVB light (311 nm, TL01) twice per week. The genital area was always shielded during the treatment, and patients were provided with UVB-blocking goggles to protect their eyes. After UVB exposure, patients were advised to use topical emollients. Side effects were monitored and documented during each visit, including erythema (skin redness), burning of the skin, hyperpigmentation, and blistering. A questionnaire was employed to document the extent, intensity, frequency, and sleep disruption experienced to evaluate the efficiency of the treatment, using a scale from 0 to 10.

This research had favorable consent from the Marrakesh University Hospital Ethics Committee (reference number 12/2017). This study was conducted according to the guidelines of the Declaration of Helsinki. The objectives of the study were explained to the participants, and voluntary informed consent was obtained from all patients. All information collected from the participants was kept confidential.

## Results

In a group of 38 patients, the average age of the patients was 56 years (16-80); 63.2% were female and 36.8% were male. The median duration of pruritus was 4.7 years (one month to 17 years), and that of dialysis was 8.4 years (three months to 25 years). Pruritus was intermittent and diffuse in most cases, localized to the arteriovenous fistula site in two cases, and exacerbated by heat in all cases. Itch intensity was evaluated with a numerical rating scale (0-10) based on the worst level of itching in the past two weeks and showed a moderate average score (5/10).

All patients gave a history of using antihistamines; 22 patients received topical emollients; two patients received steroids; and one patient received gabapentin, but the response was unsatisfactory in all patients. The main clinical and laboratory findings of our study group are detailed in Table 1.

**Table 1:** Clinical and laboratory findings in the studied patients. BMI: body mass index, HD: hemodialysis, Hb: hemoglobin, Hct: hematocrit, Alb: albumin, AST: aspartate aminotransferase, ALT: alanine aminotransferase, PTH: parathormone, CRP: c-reactive protein, TSH: thyroid-stimulating hormone.

	Value
Age (years), mean	56 years
Male/female, n	24/14
BMI (kg/m^2^), mean	20
Associated comorbidities, n (%)	
Diabetes	34%
Hypertension	30%
Coronary artery	22%
Hemodialysis data, median	
HD duration (months)	100 months
Kt/v	1.24
Biochemical data median (minimum-maximum)	
Hb (mg/dl)	10.2 (8.9-14)
HCT (%)	33 (29-39)
Alb (mg/dl)	33 (29-45)
AST (U/L)	11 (8-22)
ALT (U/L)	16 (8-30)
Calcium (mg/dl)	8.65 (7.2-9.9)
Phosphorus (mg/dl)	4.5 (2.1-6.2)
PTH (pg/ml)	327 (65-502)
Ferritin (ng/ml)	441 (127-650)
CRP	5 (1-15)
TSH (µIU/ml)	3.3 (1.13-4.5)

Xerosis was found in 63%, and scratch lesions such as excoriation in 34%. NB-UVB phototherapy was used twice per week on nonconsecutive days, with protection of the genital area and also the eyes using UVB-blocking goggles. The initial dose was 0.4 J/cm^2^, and further doses were introduced according to the erythema response until a maximum of 2 J/cm^2^. No sunburn, hyperpigmentation, or blistering was noted. Emollients were maintained in patients with xerosis. The average number of sessions was 13 (6-24), and a reduction of itch intensity was observed starting from the sixth session. Total improvement was obtained at the end of the treatment duration, except for three patients who required additional sessions. One patient had a recurrence one year later. 

**Figure 1:**
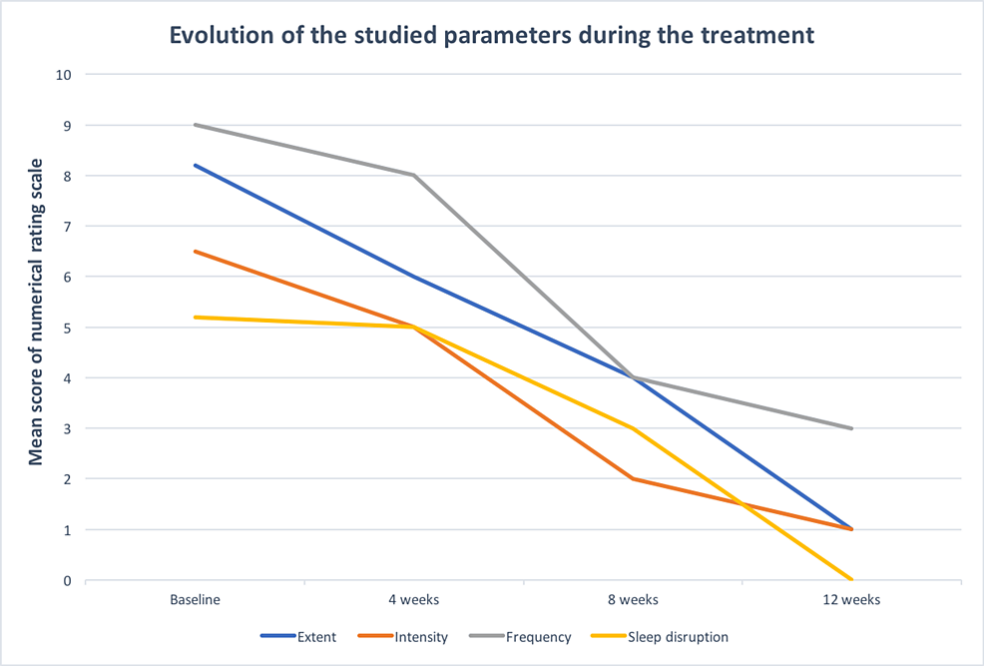
Pruritus severity in patients before and after treatment.

There was a tendency of higher ratings of itching severity on the numerating rating scale in older age, longer duration of renal disease, and longer duration of pruritus. The severity of pruritus in patients before and after treatment is shown in Figure 1, and a comparison of biological parameters is stated in Table 2.

**Table 2:** Comparison of patients before and after treatment in terms of parameters, including calcium, phosphorous, PTH, and hematocrit. As demonstrated in the table, no significant difference was seen before and after treatment regarding variables including calcium, phosphor, PTH, and Hct. PTH: parathormone, Hct: hematocrit.

Variables	Median before treatment	Median after treatment
Calcium (mg/dl)	8.65	8.52
Phosphorus (mg/dl)	4.5	3.9
PTH (pg/ml)	327	405
Hct (%)	33	37

## Discussion

Chronic kidney disease (CKD) affects millions of individuals worldwide, and one of its most distressing and pervasive symptoms is pruritus, or severe itching [1]. While traditional treatments have provided limited relief, the emergence of phototherapy, particularly narrowband ultraviolet B (NB-UVB) phototherapy, is bringing new hope to those suffering from chronic kidney disease-associated pruritus (CKDaP) [2].

The efficacy of phototherapy in managing chronic kidney disease-associated pruritus (CKDaP) has been a subject of interest and research. Studies have shown that NB-UVB phototherapy can significantly reduce pruritus in CKD patients, offering them much-needed relief. It acts as a glimmer of hope for those who have struggled to find solutions through conventional treatments [3,4].

While the precise mechanisms through which phototherapy alleviates pruritus are not fully understood, it is believed to work by modulating the immune system and reducing the inflammatory responses that contribute to itching [5]. What's clear is that the results have been promising, as multiple studies have reported reduced itching and improved quality of life in patients [4].

In our current investigation, we have successfully showcased the effectiveness of NB-UVB therapy in mitigating chronic kidney disease-associated pruritus (CKDaP). The majority of our patients presented with moderate to severe pruritus, as indicated by their numerical rating scale score, which ranged from four to 10. Notably, these patients had previously shown resistance to standard medical treatments.

After undergoing six UVB sessions, we observed a significant reduction in the numerical rating scale scores, and this improvement continued to steadily increase with successive treatments. By the end of the study, a noteworthy 93% of patients were classified as responders, having achieved an 85% reduction in their intensity itching scores compared to the baseline [8-13].

In our study, the median hemoglobin levels (10.2 mg/dl) are very near the target set by KDIGO [6]. The serum phosphate levels (median value 4.5 mg/dL) and intact parathyroid levels (median value 327.28 pg/mL) were also reasonable for patients on dialysis. All patients who completed the study had adequate kt/v >1.4, suggesting that factors other than inadequate dialysis played a significant role in the pathogenesis of uremic pruritus in our patients.

The small sample size, short duration of follow-up, and lack of blinding were the limitations of our study. The results demonstrate that narrowband ultraviolet B phototherapy may be considered an effective therapeutic option for the treatment of intractable chronic kidney disease-associated pruritus (CKDaP). Better-designed studies are needed to confirm its long-term efficacy and safety.

However, the effectiveness of phototherapy in addressing chronic kidney disease-associated pruritus (CKDaP) has already been the subject of numerous prior studies. For example, Ada et al. treated 20 Turkish patients with chronic kidney disease-associated pruritus (CKDaP), with 90% having Fitzpatrick phototype 3 or higher. They reported a substantial improvement in the visual analog scale (VAS) and pruritus scaling system after a six-week treatment period, with 80% of cases displaying positive responses. However, a recurrence of symptoms was noted in 57% of patients six months later [3]. Seckin et al. examined 17 chronic kidney disease-associated pruritus (CKDaP) patients using a similar scoring system, with 67% having Fitzpatrick phototype 3. They reported a remarkable improvement in pruritus in 60% of cases after eight weeks, but with a 66% relapse rate [8]. More recently, Wang et al. conducted a case-controlled study, revealing a significant improvement in pruritus intensity in the NB-UVB-treated group (68.4%) compared to the control group [9,10].

Our findings align with these previous studies. Not all CKD patients with pruritus are suitable candidates for phototherapy. Individualized assessments are necessary to determine the most appropriate treatment plan for each patient, taking into consideration the severity of the disease, any potential contraindications such as skin cancer occurrence, and other individual factors [12,13].

Exploring the potential of combining phototherapy with other treatment modalities, such as emollients, may hold the key to maximizing the effectiveness of pruritus management. This approach could offer a well-rounded solution for patients [12].

## Conclusions

In conclusion, phototherapy, particularly NB-UVB, represents a significant advancement in the treatment options for CKD-associated pruritus. Its positive impact on reducing itching and improving the quality of life for many patients is undeniable. However, to fully unlock its potential, ongoing research is needed to optimize dosing, understand relapse mechanisms, and identify the patients who will benefit most from this therapy. Phototherapy should be considered in conjunction with other treatment options, and its role in the management of CKD-associated pruritus is poised to evolve as more data becomes available. It is a ray of hope in the fight against a persistent and burdensome symptom, bringing comfort and relief to those who need it most.
